# mTORC1-Rps15 Axis Contributes to the Mechanisms Underlying Global Translation Reduction During Senescence of Mouse Embryonic Fibroblasts

**DOI:** 10.3389/fcell.2019.00337

**Published:** 2019-12-11

**Authors:** Su Wu, Siyao Xu, Ruofei Li, Kecheng Li, Xiaoqin Zhong, Yingying Li, Zhifen Zhou, Yi Liu, Ran Feng, Jianfei Zheng, Zhou Songyang, Feng Liu

**Affiliations:** MOE Key Laboratory of Gene Function and Regulation, Institute of Healthy Aging Research, School of Life Sciences, Sun Yat-sen University, Guangzhou, China

**Keywords:** cell senescence, MEF, mRNA translation, mTORC1, Rps15

## Abstract

The reduction of protein translation is a common feature in senescent cells and aging organisms, yet the underlying mechanisms are not fully understood. Here we show that both global mRNA translation and mammalian/mechanistic target of rapamycin complex 1 (mTORC1) kinase activity are declined in a senescent model of mouse embryonic fibroblasts (MEFs). Furthermore, RNA-seq analyses from polysomal versus total mRNA fractions identify TOP-like mRNA of *Rps15* whose translation is regulated by mTORC1 during MEF senescence. Overexpression of *Rps15* delays MEF senescence, possibly through regulating ribosome maturation. Together, these findings indicate that the activation of mTORC1-Rps15 axis ameliorate senescence by regulating ribosome biogenesis, which may provide further insights into aging research.

## Introduction

Cell senescence is a state of irreversible cell cycle arrest, accompanying with a gradual degeneration of physiological functions during the passage of time (He and Sharpless, 2017; Lee and Schmitt, 2019). In the year of 1961, Dr. Leonard Hayflick and Paul Moorhead firstly observed that human fibroblasts have finite proliferation capacity in culture, which is further termed senescence (Hayflick and Moorhead, 1961). Since then, mammalian fibroblasts have been widely used in establishing senescence model for aging research by serial cultivation *in vitro*. Cell senescence can be triggered by many stresses, including telomere shortening, genomic damage, oncogene activation or oxidative stress, which are factors also contribute to organismal aging or aging-related diseases (Childs et al., 2015; De Magalhaes and Passos, 2018; McHugh and Gil, 2018). Therefore, uncovering the mechanism, especially the intrinsic mechanism of cell senescence will help to meet the challenge of aged population worldwide.

During cell senescence, some hallmarks are regarded as aging driving forces, among which proteostatic dysfunction is caught much attention in recent years (Lopez-Otin et al., 2013; Hernandez-Segura et al., 2018; McHugh and Gil, 2018). Proteostasis is a state of properly balanced proteome involving protein synthesis, maturation and degradation (Hipp et al., 2019). Growing evidences suggest that ribosomes act as hub for protein translation, co-translational folding and even protein degradation (Sherman and Qian, 2013). Therefore, how to precisely control ribosomal behaviors, including protein translation within the cell, is very important for proteostasis maintenance and anti-aging.

Recently, the significance of mRNA translation as a key regulator of human diseases has been rapidly increased, including in aging-related research (Anisimova et al., 2018; Tahmasebi et al., 2018). More evidences support the idea that protein synthesis is decreased during aging in a wide variety of cells; and alteration of the translation machinery affecting the rate and selectivity of protein biosynthesis seem to play a central role in cell senescence and organismal aging (Gonskikh and Polacek, 2017). Hence, polysomal profiling strategy is becoming a regular technique in molecular biology lab to study the mechanism regulating mRNA translation process (Chasse et al., 2017). Using sucrose gradient ultracentrifugation to separate actively translated mRNAs which bound by several ribosomes from ribosomal subunits and monosomes enables researcher to investigate ribosomal assembly and mRNA translation regulation. After isolated from sucrose gradient, polysomal-mRNAs and total mRNA can be analyzed by various methods including RNA-seq to study mRNA translation process (King and Gerber, 2016).

Mammalian/mechanistic target of rapamycin complex 1 (mTORC1) is a serine/threonine protein kinase for regulating mRNA translation (Saxton and Sabatini, 2017; Roux and Topisirovic, 2018). Upon activation, mTORC1 phosphorylates its downstream substrate, ribosomal protein S6 kinase 1 (S6K1) at T389. Activated S6K1 phosphorylates and activates mRNA translation initiation factor eukaryotic translation initiation factor 4B (eIF4B) for the formation of 5′ cap binding eIF4F complex (Holz et al., 2005). Eukaryotic translation initiation factor 4E binding protein 1 (4E-BP1) is another mTORC1 substrate that inhibits translation by sequestrating eIF4E (Brunn et al., 1997). After phosphorylation by mTORC1 at multiple sites including T37, T46, S65, and T70, hyper-phosphorylated 4E-BP1 dissociates from eIF4E, thus allowing the formation of the 5′ cap binding eIF4F complex (Gingras et al., 1999).

Although many researchers have noticed that mRNA translation is declined during senescence, how mRNA translation and ribosome biogenesis regulate senescent process is to be elucidated. In this study, we tried to use polysomal profiling and RNA-seq technology to discover the underlying mechanisms of mRNA translation reduction in senescent mouse embryonic fibroblasts (MEFs). We show that global mRNA translation is decreased in a senescence model of MEFs. We further characterize that mTORC1 pathway regulates TOP-like *Rps15* mRNA translation, which contributes to the ribosome biogenesis and finally ameliorate cell senescence.

## Results

### Protein Synthesis Is Globally Reduced in Senescent MEFs

**FIGURE 1:**
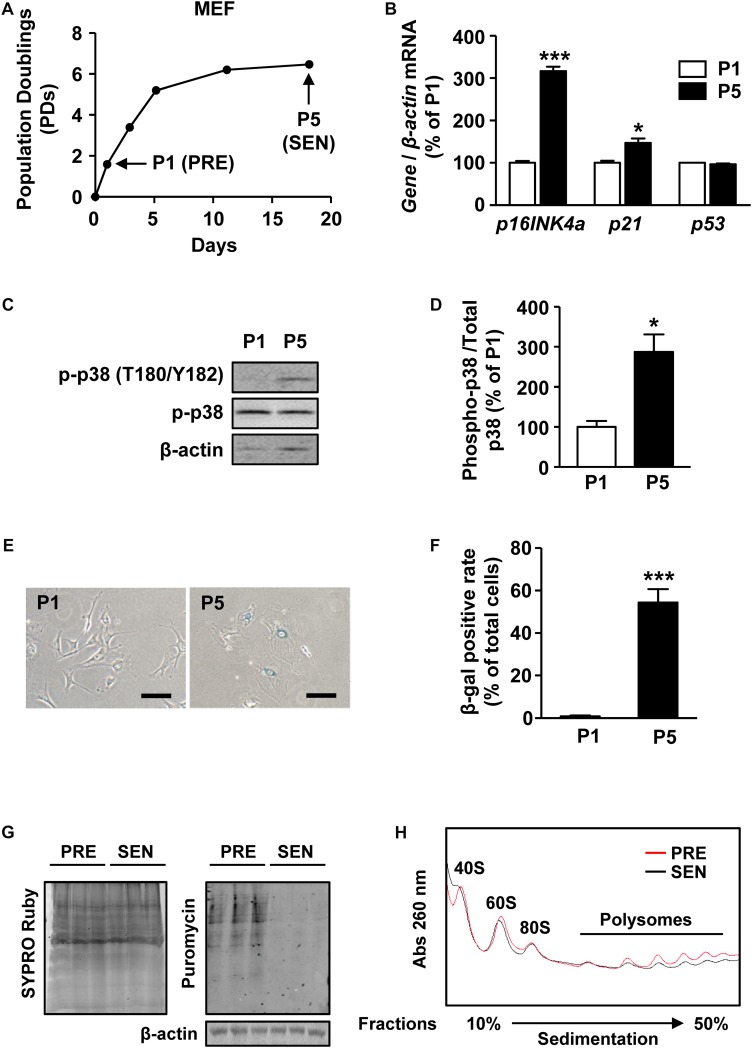
Protein synthesis is globally reduced in senescent MEFs. **(A)** Growth curve of MEFs from passage 0 to passage 5. PRE and SEN refer to presenescent and senescent, separately. **(B)** Relative quantification of *p16INK4a*, *p21*, and *p53* mRNAs in young and senescent MEFs. β*-actin* was used as internal control (Mean ± SEM, *n* = 3, ^∗∗∗^*p* < 0.001, ^∗^*p* < 0.05). **(C,D)** Representative western blot and quantification of phosphorylated protein and total protein levels of p38 in cell extracts from passage 1 and passage 5 MEFs. Total p38 protein was used as internal loading control (Mean ± SEM, *n* = 3, ^∗^*p* < 0.05). **(E,F)** SA-β-gal staining and SA-β-gal positive rate of MEFs in passage 1 and passage 5 (Mean ± SEM, *n* = 3, ^∗∗∗^*p* < 0.001). **(G)** Puromycin incorporation assay of presenescent and senescent MEFs. SYPRO Ruby staining was used to visualize total proteins (left) and immunoactivity of puromycin indicates nascent peptide synthesis rate (right). β-actin was used as internal loading control. **(H)** Polysomal profiles of young and senescent MEFs with continuous sucrose gradient of 10–50% were fractioned and measured with absorbance of light at 260 nm. Peaks belonged to small subunit of 40S, large subunit of 60S, intact ribosome of 80S and polysomes were labeled.

Cultured MEFs are widely used to study the mechanism of cell senescence (Parrinello et al., 2003; Di Micco et al., 2008). Here we established a senescent MEF model by continuously passaging. When cultured *in vitro*, MEFs experienced accelerated cell growth arrest as early as passage 5 (P5) (Figure 1A). Compared with presenescent (PRE) MEFs at passage 1 (P1), P5 MEFs expressed significantly higher level of aging markers *p16INK4a* and *p21* (Figure 1B). Mitogen-activated protein kinase p38 activation is a hallmark of stress-induced MEF senescence (Iwasa et al., 2003). Increased phosphorylation of p38 in P5 MEFs reflects the senescence (Figures 1C,D). In addition, β-gal staining results showed that more P5 MEFs were flattened (Figure 1E) and stained as β-galactosidase positive than P1 cells (Figure 1F), indicating that P5 MEFs underwent senescent and thus were used as senescent cells (SEN) in the subsequent studies.

To test whether protein synthesis alters during senescence, we performed puromycin incorporation assay of surface sensing of translation (SUnSET) (Schmidt et al., 2009), a non-radioactive method to analyze protein synthesis in P1 and P5 MEFs. By mimicking transfer RNA (tRNA), puromycin can be incorporated into nascent peptide and further detected by western blot analysis with anti-puromycin monoclonal antibody. The immunoactivity of puromycin was decreased in SEN group, suggesting that less puromycin was conjugated within proteins in SEN group compared with PRE group for the same labeling time of 10 min (Figure 1G). Therefore, the protein synthesis was lower in senescent MEFs.

Polysome profiling is a method to analyze translating mRNAs according to the number of bound ribosomes separated on a sucrose gradient. We found that the overall polysome abundance in senescent cells was lower than in presenescent cells, suggesting that a global decrease of protein translation occurs during senescence (Figure 1H). Several factors, including ribosome biogenesis, ribosome subunit assembly, formation of translation initiation complexes, could contribute to mRNA translation process. However, we also noticed a slightly reduction of 80S peak as wells as ribosomal subunit peaks appeared before 80S peak in light fractions of senescent MEFs (Figure 1E), meaning a declined number of intact ribosomes during senescence.

Together, these results raised a possibility that decreased ribosome biogenesis may underlie the mechanism of global translation reduction in senescent MEFs.

### Polysomal RNA-seq Reveals That the Ribosome Biogenesis Is Deficient in Senescent MEFs

**FIGURE 2:**
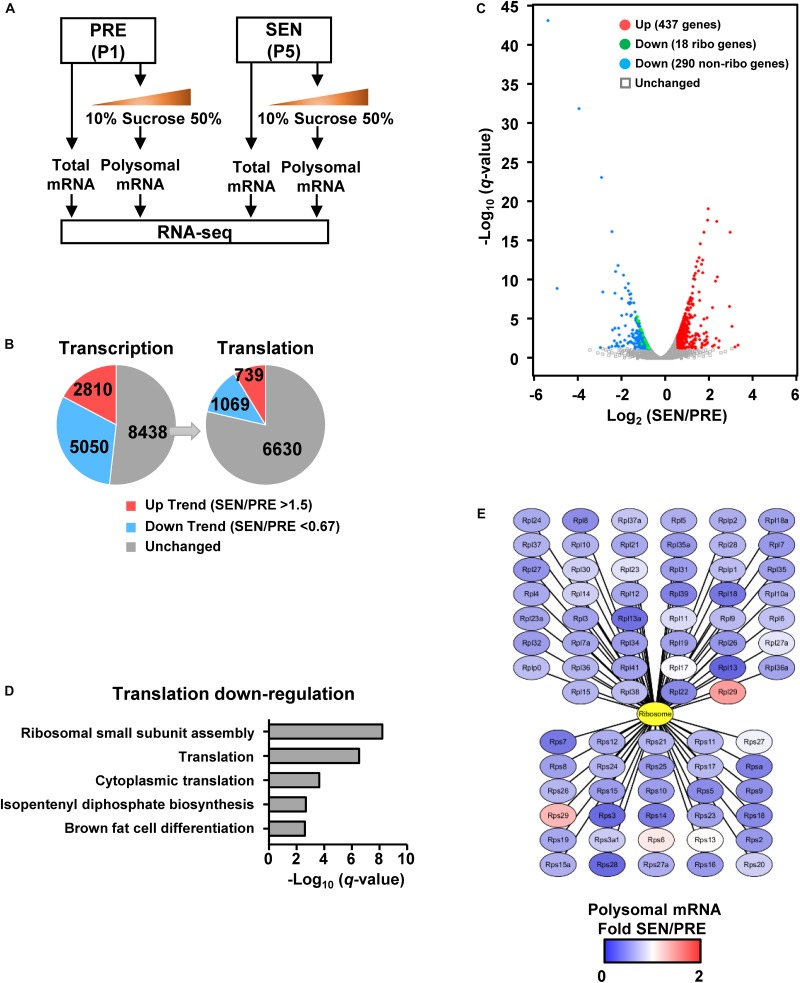
Polysomal RNA-seq reveals that the ribosome biogenesis is deficient in senescent cells. **(A)** RNA-seq samples are collected using total mRNA extract and polysomal mRNA fraction in young and senescent MEFs. **(B)** Gene numbers of transcriptional and translational changes were calculated from comparing total and polysomal RNA-seq data. The threshold of fold-changes was set to 1.5 for the trend of up-regulation and 0.667 for the trend of down-regulation. **(C)** Volcano plot shows significantly regulated genes on translational level. The threshold of significance was set to FPKM SEN/PRE >1.5, FDR *q* < 0.05 for up-regulation and FPKM SEN/PRE <0.67, FDR *q* < 0.05 for down-regulation. **(D)** Biological process analysis of genes which were only down-regulated in translational level shown in panel **(C)**. **(E)** Translationally changed genes of ribosomal protein calculated from polysomal RNA-seq data. All 46 large subunit ribosomal proteins were illustrated in the upper part, while 30 small subunit ribosomal protein were shown in the lower part.

To further study what aspects of the cell are affected due to global translation reduction during cell senescence, we firstly performed RNA-seq without replicates in both presenescent and senescent cells, aiming to compare total and polysomal RNA levels. Half of P1 and P5 MEF samples were subjected to total mRNA isolation, and RNA-seq results of the total mRNA revealed differential gene expression on their transcriptional levels during senescence (Figure 2A). Meanwhile, the other half of each samples were loaded on sucrose gradients for polysome profiling. The RNA-seq results of polysomal mRNA fraction reflects mRNA translational level, which would be more correlated with protein levels (Figure 2A). To minimize the number of transcriptionally unchanged genes, we firstly set a lower threshold to 1.5 without consideration of *q*-value between groups and found trends that 2810 genes were up-regulated [fragments per kilobase of transcript per million mapped fragments (FPKM) of SEN/PRE >1.5], while 5050 genes were down-regulated (FPKM of SEN/PRE <0.67) on the transcriptional levels in senescent cells (Figure 2B and Supplementary Table S1). Gene ontology (GO) analysis of these genes showed that transcriptional up-regulated genes were enriched in inflammatory response pathways, while transcriptional down-regulated genes were basically participated in DNA repair pathways (Supplementary Figure S1), which also verified the senescent state of the cells.

Among those 8438 genes whose transcriptional levels were of unchanged trend during senescence, 1069 genes were decreased by at least 0.67 fold and 739 genes were increased more than 1.5 folds on their translational levels after undergoing senescence (Figure 2B). If false discovery rate (FDR) is taken into account, 437 genes were significantly up-regulated (FPKM of SEN/PRE >1.5, FDR *q* < 0.05) and 308 genes were significantly down-regulated (FPKM of SEN/PRE <0.67, FDR *q* < 0.05) (Figure 2C and Supplementary Table S2).

Given that the global translation is reduced during senescence (Figures 1G,H), we focused on translationally down-regulated genes. GO analysis revealed that those significantly down-regulated genes on translational level were enriched in cytoplasmic translation as well as ribosomal small subunit assembly (Figure 2D). Besides, 18 genes encoding ribosomal proteins were significantly down-regulated on translational level (Figure 2C) and almost all of the ribosomal proteins’ translation has a declined trend in senescent cells (Figure 2E), raising a possibility that senescence-related ribosome biogenesis deficiency might be a result of down-regulating ribosome protein translation.

### mTORC1 Regulates *Rps15* mRNA Translation During Senescence

**FIGURE 3:**
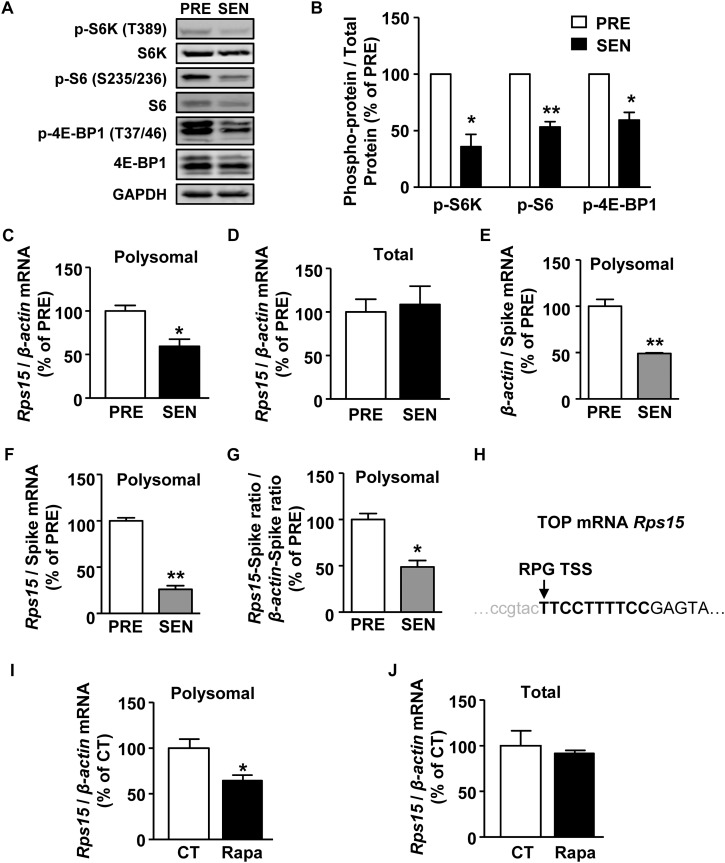
mTORC1 regulates *Rps15* mRNA translation during senescence. **(A,B)** Representative western blot and quantification of phosphorylated protein and total protein levels of S6K, S6, 4E-BP1 in cell extracts from young and senescent MEFs. Relative total proteins were used as internal loading control (Mean ± SEM, *n* = 3, ^∗∗^*p* < 0.01, ^∗^*p* < 0.05). **(C,D)** Relative quantification of *Rps15* mRNA using polysomal mRNAs **(C)** and total mRNAs **(D)** extracted from young and senescent MEFs. β*-actin* was used as internal control (Mean ± SEM, *n* = 3, ^∗^*p* < 0.05). **(E,F)** Relative quantification of β*-actin* **(E)** and *Rps15* **(F)** mRNA using polysomal mRNAs extracted from young and senescent MEFs with adding spike RNA of mScarlet after polysome profiling. Spike RNA of mScarlet mRNA was used as internal control (Mean ± SEM, *n* = 3, ^∗∗^*p* < 0.01). **(G)** Relative quantification of *Rps15* mRNA-spike ratio to β*-actin*-spike RNA ratio from the results of panels **(A,B)** (Mean ± SEM, *n* = 3, ^∗^*p* < 0.05). **(H)** Transcription start site (TSS) annotations for TOP-like mRNA *Rps15*. Arrow head indicates TSS. Bold letters refer to TOP-like structure. *Rps15* 5’UTR sequence was from Ribosomal Protein Gene Database (RPG). **(I,J)** Relative quantification of *Rps15* mRNA using total mRNAs **(I)** and polysomal mRNAs **(J)** extracted from DMSO or rapamycin treated young MEFs (250 nM, 2 h). β*-actin* was used as internal control (Mean ± SEM, *n* = 3, ^∗^*p* < 0.05).

Next, we investigated in the signaling pathway regulating senescence-related ribosome biogenesis deficiency. mTORC1 is a serine/threonine protein kinase which primarily regulates translation initiation and plays an important role in cell senescence (Saxton and Sabatini, 2017). Activated mTORC1 phosphorylates its two substrates, p70 S6 kinase 1 (S6K) and eIF4E binding protein (4E-BP), promoting downstream translation of 5′ terminal oligopyrimidine structure (TOP) or TOP-like mRNAs, which contains ribosomal protein mRNA (Hsieh et al., 2012; Thoreen et al., 2012). S6K can also regulate ribosomal biogenesis and assembly by further phosphorylates ribosomal protein S6 (Chauvin et al., 2014). As shown in Figures 3A,B, the phosphorylation of S6K, S6, and 4E-BP significantly decreased in senescent cells, suggesting declined mTORC1 activity during MEF senescence.

5′ terminal oligopyrimidine structure structure is a stretch of 4–14 pyrimidines immediately after the transcription start site (TSS) of certain mRNAs, of which translation are thought to be regulated by mTORC1 (Thoreen et al., 2012). To identify mTORC1-regulated mRNA during senescence, we used an *in silico* approach from Refseq^1^ or Ribosomal Protein Gene Database (RPG) (Nakao et al., 2004) and found 33 TOP mRNAs and 21 TOP-like mRNAs from 1069 translationally down-regulated genes (Supplementary Figures S2A,B and Supplementary Table S3). Huo et al. (2012) used SILAC-based approach to identify mRNAs whose translation is sensitive to mTORC1 in rapamycin treated HeLa cells. We compared their data with ours and found that 13 genes were overlapped, among which nine genes encode ribosomal proteins (Supplementary Figure S2C).

From 54 translational down-regulated TOP/TOP-like genes in RNA-seq results (Supplementary Figures S3, S4), we chose 30 genes for further qPCR confirmation for their primers were of satisfying amplification efficiency. By using quantitative reverse transcription-PCR (qRT-PCR), we confirmed that mRNA levels of five genes, including *Rps15*, *CDK2 associated, cullin domain 1* (*Cacul1)*, *tRNA aspartic acid methyltransferase 1* (*Trdmt1)*, *Zinc finger protein 280D* (*Zfp280d*), and *HAUS augmin-like complex, subunit 1* (*Haus1*), showed a decrease (<0.67 fold) in the polysomal mRNA fraction of senescent MEFs (Figure 3C and Supplementary Figure S5A), while their corresponding mRNA levels remained unaltered in the total mRNA fractions (Figure 3D and Supplementary Figure S5B). To normalize the mRNA association with polysomes, we added a spike RNA in each fraction of the sucrose gradient before purifying polysomal RNA. Both β*-actin* and *Rps15* mRNA levels were dramatically decreased during senescence in polysomal fractions when normalized with exogenous spike RNA, suggesting a global translation reduction (Figures 3E,F). However, *Rps15* mRNA showed even greater reductions than β*-actin* mRNA, which confirmed results without adding spike RNA in Figure 3C (Figure 3G).

Given these five candidate mRNAs contains either TOP or TOP-like structures (Figure 3H and Supplementary Figure S2B), we hypothesized that mTORC1 regulates translation of these mRNAs. To test this possibility, we treated presenescent MEFs with an mTORC1 inhibitor, rapamycin. As shown in Figures 3I,J, 2-h treatment of 250 nM rapamycin significantly reduced only *Rps15* mRNA in the polysomal fraction but not in the total fraction. While, the mRNA levels of other candidates, *Cacul1*, *Trdmt1*, *Zfp280d*, and *Haus1*, in polysomal fractions remained unaffected by rapamycin (data not shown). Together, these results suggest that decreased activity of mTORC1 down-regulates a TOP-like mRNA of *Rps15* translation during senescence.

### Rps15 Is Important for Ribosome Biogenesis and Overexpression of *Rps15* Ameliorates Senescent Phenotypes in MEFs

**FIGURE 4:**
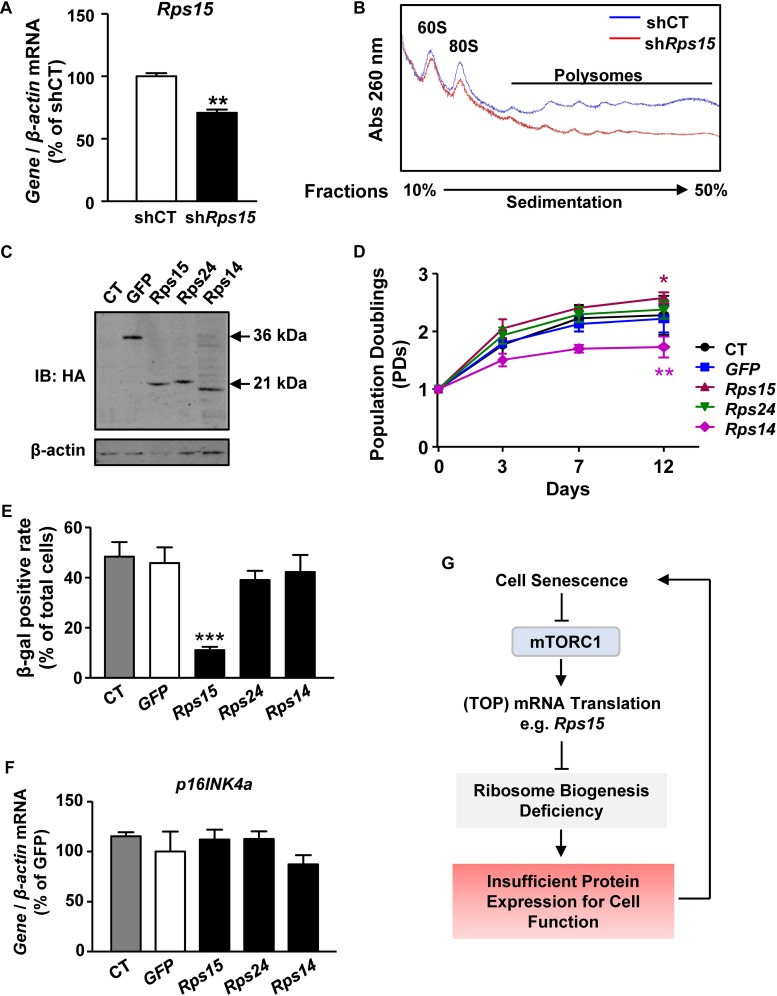
*Rps15* is important for ribosome biogenesis and overexpression of *Rps15* ameliorates senescent phenotypes in MEFs. **(A)** Relative quantification of *Rps15* mRNA after knockdown *Rps15* in MEFs. β*-actin* was used as internal control (Mean ± SEM, *n* = 3, ^∗∗^*p* < 0.01). **(B)** Polysomal profiles of sh*Rps15* MEFs and negative control with continuous sucrose gradient of 10–50% were fractioned and measured with absorbance of light at 260 nm. Peaks belonged to large subunit of 60S, intact ribosome of 80S and polysomes were labeled. **(C)** Representative western blot analysis of untransfected control MEFs (CT) and overexpression of *GFP*, *Rps15*, *Rps14*, and *Rps24* in extracts from respective stable-expression MEFs, recognized by anti-HA (mouse). β-actin was used as internal loading control. **(D)** Growth curves of *Rps15*, *Rps24*, *Rps14*, *GFP* stable transgenic MEFs and untransfected control MEF (CT) starting from passage 1 (Mean ± SEM, *n* = 3, ^∗^*p* < 0.05, ^∗∗^*p* < 0.01). **(E)** SA-β-gal positive rate of senescent *Rps15*, *Rps24*, *Rps14*, *GFP* stable-expression MEFs and untransfected control MEFs (CT) (Mean ± SEM, *n* = 3, ^∗∗∗^*p* < 0.001). **(F)** Relative quantification of *p16INK4a* mRNAs in senescent *Rps15*, *Rps24*, *Rps14*, *GFP* stable-expression MEFs and untransfected control MEFs (CT). β*-actin* was used as internal control (Mean ± SEM, *n* = 3). **(G)** A schematic model of mTORC1-Rps15 axis contributes in senescence-associated translation reduction.

As a ribosomal small subunit protein, Rps15 is essential for the final maturation and assembly of ribosome 40S subunit (Robledo et al., 2008). Therefore, we presumed that declined translation of *Rps15* mRNA leads to defect ribosome biogenesis and cell senescence. To test this hypothesis, we firstly transiently knocked down *Rps15* mRNA in presenescent MEFs by lentiviral short hairpin RNA expression (Figure 4A and Supplementary Figures S6A,B). Knockdown of *Rps15* mRNA did not affect other ribosomal mRNA transcription, such as *Rps24* (Supplementary Figure S6C). Polysome profiling experiments showed that knockdown *Rps15* strongly inhibited the 80S peak in MEFs, which partially mimics the assembly deficiency of 80S subunit in senescent MEFs (Figure 4B), suggesting that the decreased expression of *Rps15* might contribute to ribosome biogenesis deficiency in senescent MEF cells.

*Rps14* encodes another ribosomal small subunit protein, whose translation is also decreased during MEF senescence as shown in RNA-seq results (Figure 2C). In contrast, the translation *Rps24* did not change during MEF senescence (Supplementary Table S2). To further test the role of *Rps15* in cell senescence, we overexpressed *Rps15* in presenescent MEFs, while the ectopic expression of *GFP*, *Rps14*, and *Rps24* were used as controls (Figure 4C). These cells were cultured from P1 until control cells underwent senescence. The growth curve showed that MEFs overexpressed *Rps15* gained more potential to proliferate even after control cells stopped growing, while overexpression of *Rps14* inhibits cell proliferation (Figure 4D). We also noticed a decreased β-gal positive rate in MEFs only after overexpression of *Rps15* (Figure 4E). However, the *p16INK4a* mRNA levels showed no differences among groups (Figure 4F), indicating that Rps15 acts in a p16INK4a-independent pathway to ameliorate MEF senescence. Unlike *Rps15*, knockdown of *Rps14* in presenescent MEFs did not induce a significant translation reduction, suggesting that the role of Rps15 in regulating MEF senescence is gene specific (Supplementary Figures S6D,E).

Eukaryotic translation initiation factor 4E binding protein 1 is a downstream target of mTORC1 (Brunn et al., 1997). Upon phosphorylation by mTORC1, 4E-BP1 allows eukaryotic translation initiation factor (eIF4E) to be released and activated for TOP mRNA translation (Gingras et al., 1999). Knockdown of *4E-BP1* in senescent MEFs by lentivirus-mediated shRNA delivery inhibited *p16INK4a* expression but did not affect *p21*, *p53*, and *Rps15* expression on transcriptional levels (Supplementary Figure S7A). Effective *4E-BP1* knockdown could not change Rps15 protein levels (Supplementary Figures S7B,C). Moreover, the senescent MEF proliferation as well as the percentage of β-gal staining could not be rescued (Supplementary Figures S7D,E), suggesting that targeting *4E-BP1* is not sufficient to mimic overexpression of *Rps15* in delaying MEF senescence.

## Discussion

Cell senescence is an irreversible non-proliferative state, which can be triggered by different cell stresses, including oxidative stress in MEFs cultured regularly *in vitro* (Parrinello et al., 2003). Proteostasis, namely protein homeostasis, is a relatively steady process involved protein biogenesis, maturation and degradation, which is important for homeostatic maintenance within a cell (Klaips et al., 2018). Loss of proteostasis is one of the common features as well as drivers of aging, because either excessive misfolded protein-induced protein aggregation or insufficient protein expression is harmful to the normal function of cells (Hipp et al., 2019). Therefore, targeting proteostasis pathways is theoretically promising for aging prevention.

A key point for regulating proteostasis is ribosome-mediated mRNA translation, which largely depends on matured ribosomes. Insufficient or mutation of ribosome proteins has been documented in human diseases called ribosomopathies (Tahmasebi et al., 2018), indicating that ribosomal quality control is vital for health. In this study, we found that global translation is decreased during MEF senescence. More importantly, polysomal RNA-seq from presenescent and senescent cells revealed that the most significant enriched translationally down-regulated genes in senescent cells are those encoding proteins for assembling translation machinery, especially ribosomal proteins. These results suggested a positive feedback loop that declined mRNA translation acts on translation apparatuses *per se* to limit overall protein expression during senescence. Given that hyperactive translation-induced misfolded protein aggregates result in senescence in some cell types, our results provide another possibility that, just on the contrary, insufficient functional protein expression also occurs during senescence in certain cell types, and may lead to cell functional deficiency and senescence (Figure 4G).

Intact mammalian 80S ribosome is composed of 40S small subunit and 60S large subunit. Both of these two subunits are separately assembled in nucleus and exported into cytoplasm for mRNA translation (Tahmasebi et al., 2018). Specifically, ribosomal 40S small subunit is firstly loaded onto mRNA immediately after the formation of translation initiation complex. Next, ribosomal 60S large subunit joined with the 40S-mRNA complex, thus starting translation elongation (Gebauer and Hentze, 2004). Therefore, ribosomal 40S small subunit is key for the intact 80S ribosome assembly and mRNA translation process. In this study, we identified Rps15 plays a role in regulating senescence-associated translation reduction, which is coincide with previous study that Rps15 is vital for ribosomal 40S small subunit assembly. Rps15 is an essential component of 40S subunit of ribosome and participate in translation process, and critical for nuclear export of small subunit pre-particles (Leger-Silvestre et al., 2004). This is further supported by our GO results of genes down-regulated in translation level, which shows cytoplasmic translation was especially affected in senescent MEF. Because translation is happened mainly in cytoplasm, we reasoned that ribosome transportation and assembly would be impaired due to Rps15 reduction, finally led to cell growth arrest. However, we noticed that Rps15 knockdown does not change the 60S peak, which is not totally resembled the polysomal profiling of senescent MEF cells. It is possible that other ribosomal large subunit proteins also contribute to the declined translation during cell senescence, which requires further investigation.

Senescent cells usually express several senescent markers to indicate its non-dividing state (Hernandez-Segura et al., 2018). Most of these markers belong to cell cycle inhibitors. *p16INK4a* is one of these well-known senescent markers and has been found up-regulated in several cell types (Alcorta et al., 1996; Krishnamurthy et al., 2004). In our results, overexpression of Rps15 in MEFs could delay senescent process, yet the *p16INK4a* mRNA levels remains unaltered. These data suggest that Rps15 mediated amelioration of senescence is p16INK4a-independent and may be through improving organelle function within the cells.

The long-term use of rapamycin has been shown to extend both lifespan and healthspan in almost all the model organisms, including yeast, worms, flies and mice (Powers et al., 2006; Harrison et al., 2009; Bjedov et al., 2010; Robida-Stubbs et al., 2012). Since the rapamycin is an mTORC1 specific inhibitor and adding rapamycin also impaired ribosome biogenesis (Iadevaia et al., 2012), some researchers believe that mTORC1 activity and ribosome content should be, in theory, up-regulated during senescence. Actually, the change of mTORC1 activity in senescent process is still debated. In our model, we found a declined mTOR signaling activity, which implies the aging mechanism in our cell model was more likely to be reactive oxygen species (ROS)-centric rather than mTOR-centric senescence (Parrinello et al., 2003). Based on the present study as well as other researchers’ data, senescent cells may gradually lose the ability to reactivate mTORC1 by various stimuli rather than simply enhancing or inhibiting mTORC1 activity, although we observed a declined mTORC1 activity during senescence. The fact that continuously treatment by rapamycin starting from presenescent cells or young organism could relieve aging and extend lifespan also support this idea. Therefore, early treatment of rapamycin may restrain the amplitude of mTORC1 activation responded to stimuli and preserve mTORC1 activation ability as long as the cells or organism aged, thus finally relief aging phenotype.

In our reverse transcription-quantitative polymerase chain reaction (RT-qPCR) verify data, the translation level of *Cacul1*, *Rps15*, *Trdmt1*, *Zfp280d*, and *Haus1* were confirmed to be down-regulated in senescent MEFs, but only *Rps15* is down translated in rapamycin treated MEFs in our test. How other candidates contributes to senescence will be our future interest. *Cacul1* was found to down-regulate p53 transcriptional activity, regulation oxidative pressure and apoptosis (Kong et al., 2013; Kigoshi et al., 2015; Fukuda et al., 2017), which suggest Cacul1 may act as a suppress factor in cell aging process. *Trdmt1* plays an important role in tRNA methylation to prevent tRNA cleavage into fragment, thus stabilize overall translation (Ghanbarian et al., 2016; Jeltsch et al., 2017). *Haus1* participate in centrosome cycle and spindle assembly (Gaudet et al., 2011), which was related to cell cycle. *Zfp280d* is functioned as DNA binding transcription factor (Gaudet et al., 2011). The senescence-associated roles of these genes require further investigation.

In summary, we identify mTORC1-Rps15 axis contributes to cell senescence. Future studies involving investigating the roles of other ribosomal proteins and translation regulators during aging process will shed light on basic aging research as well as the study of aging-related pathologies.

## Materials and Methods

### Cell Culture

Mouse embryonic fibroblasts obtained from (Cyagen, Suzhou, China). Cells were cultured in DMEM/F12 + GlutaMAX media (Gibco, Waltham, MA, United States) plus 10% fetal bovine serum (Excell, St. Louis, MO, United States), 100 U/mL streptomycin/penicillin (Gibco, Waltham, MA, United States), 1% 100 × NEAA (Sigma, St. Louis, MO, United States) at 37°C in a 5% CO_2_ cell incubator.

### Senescence-Associated β-gal (SA-β-gal) Staining

Cell β-gal staining was performed using Cell Senescence β-galactosidase Stain Kit (Beyotime, Shanghai, China) according to manufacturer’s instructions. Briefly, cells were seeded in 35 mm dish for 24 h, followed by fixation in room temperature for 15 min and gently wash with PBS for three times. Then, cells were incubated with staining solution (930 μL solution C, 10 μL solution A, 10 μL solution B, 50 μL X-gal solution) overnight in 37°C without exposure to oxygen. SA-β-gal positive cell rate was quantified by a modified protocol as previously described (Noren Hooten and Evans, 2017). Briefly, acquire two non-overlapped images of each sample by light microscope with 10 × objective to include >200 cells for presenescent samples and >100 cells for senescent samples. Blue SA-β-gal positive cells and total cells were counted. β-gal positive rate was calculated and expressed as the ratio of SA-β-gal positive cells to total cells. Representative image was presented along with the statistics to show the morphology of presenescent and senescent MEFs.

### Puromycin Incorporation Assay

To monitor nascent peptide synthesis, puromycin incorporation assay is an advantageous alternative to radioactive ^35^S labeling method (Schmidt et al., 2009). As previously described (Chou and Lo, 2019), 10^5^ presenescent and senescent MEFs were seeded into each well of six-well plate 16 h before exposure to 5 μg/μL puromycin for 10 min. 8 μg total proteins of each sample were loaded onto SDS polyacrylamide gel. For the visualization of total proteins, gel was stained with SYPRO^TM^ Ruby Protein Gel Stain (Invitrogen, Waltham, MA, United States) according to manufacturer’s protocol. To measure protein synthesis rate, the puromycin labeled nascent peptides was detected by western blot analysis with anti-puromycin monoclonal antibody (MABE343, Millipore, St. Louis, MO, United States).

### Western Blot Analysis

Cell extracts were separated on SDS polyacrylamide gel and blotted on nitrocellulose membranes (Millipore, St. Louis, MO, United States). Membranes were incubated with primer antibody overnight in 4°C, and hybridized with fluorescence secondary antibody for 1 h in room temperature. The list of antibodies used are as follows: anti-phospho-p38 MAPK (Thr180/Tyr182) (D3F9), anti-p38 MAPK (D13E1), anti-S6K, anti-phospho-S6K (Thr389), anti-S6, anti-phospho-S6 (Ser235/236), anti-4EBP1, anti-phospho-4EBP1 (Thr37/46) (Cell Signal Technology, Danvers, MA, United States), anti-GAPDH (Santa Cruz Biotechnology, Dallas, TX, United States), anti-Rps15 (EPR11104) (Abcam, Cambridge, United Kingdom), anti-β-actin (ab3280) (Abcam, Cambridge, United Kingdom), anti-HA (26D11) (Abmart, Shanghai, China), anti-Puromycin (12D10) (Sigma, St. Louis, MO, United States), anti-mouse 680 and anti-rabbit 800 secondary antibodies (LI-COR, Lincoln, NE, United States). ImageJ was used for optical density quantification of western blot analysis.

### Polysome Profiling and RNA Isolation

For RT-qPCR detection, 7 × 10^5^ presenescent or senescent MEFs were lysed with 1 mL polysomal lysis buffer (140 mM NaCl, 5 mM MgCl_2_, 10 mM Tris–HCl pH 8.0, 1% Triton X-100, 0.5% sodium deoxycholate (Sigma, St. Louis, MO, United States), 0.4 U/μL RNase inhibitor (Promega, Madison, WI, United States), 20 mM DTT (Sigma, St. Louis, MO, United States), 0.1 mg/mL cycloheximide, 10 mM RVC, 0.1% cocktail (Sigma, St. Louis, MO, United States) on ice for 15 min and centrifuged at 16,000 × *g* for 15 min. For total fraction, 200 μL of the supernatant was subjected to total RNA extraction using RNAiso Plus (TAKARA) reagent. The rest of supernatant was loaded onto the top of 10–50% sucrose density gradient made by density gradient pump (BioComp Instruments, Fredericton, NB, Canada). After ultracentrifuge in a SW41Ti rotor (Beckman Coulter, Brea, CA, United States) at 170,000 × *g* for 2 h, polysomal fractions were isolated with gradient fractionator (BioComp Instruments, Fredericton, NB, Canada) by continuous measurement of absorbance at 260 nm. For spike RNA incorporation assay, *in vitro* transcribed red fluorescent protein mScarlet mRNA with poly A tails was added into collected PRE or SEN polysomal fractions for a final concentration of 250 ng/mL. Same volume of RNAiso Plus (TAKARA) was added to polysomal fractions, then extracts RNA according to manufacturer’s protocol.

### RT-qPCR Detection

For mRNAs relative quantification 1 μg of RNA was reverse transcribed with PrimeScript RT Reagent Kit with gDNA Eraser (TAKARA, Shiga, Japan) according to protocol. Real time PCR was performed using ChamQ^TM^ Universal SYBR qPCR Master Mix (Vazyme, Nanjing, China). The threshold cycles (Ct) of candidate genes were normalized with *Actin*, and the result is calculated using 2^–ΔΔCt^ method. The primer sequences were listed in Supplementary Table S4.

### Cell Growth Curve Plotting

Cells were successively subcultured from passage 1, and cell number counts were conducted simultaneously. The logarithm (Log_2_) of the collected cell numbers to plated cell numbers in each plate was taken as the cell population doubling (PD), the number of PD was continuously added in each generation, and the XY coordinate function was constructed with the cell culture time to obtain the cell growth curve.

### GO Biological Process Analysis

Gene ontology biological process analysis of RNA-seq results were performed using FunRich version software 3.1.3.

### RNA Interference and Overexpression Stable Cell Line Generation

GFP and mouse *Rps15*,*Rps14*, and *Rps24* open reading frame was cloned into pLenti vector with N-terminal HA tag. *Rps15*, *Rps14*, *Rps24*, and *4E-BP1* shRNA was ligated into pLKO.1 vector (sh*Rps15* target sequence 5′-AAG TTC ACC TAC CGT GGC GTA-3′; sh*Rps14* target sequence 5′-AAG ATT GGG CGG ATT GAG GAT-3′; sh*Rps24* target sequence 5′-AAT GAG CCT AAA CAC AGA CTG-3′; sh*4E-BP1* target sequence 5′-AAC CAG GAT TAT CTA TGA CCG-3′; shCT sequence 5′- TCC GCA GGT ATG CAC GCG TGA-3′). Vectors were transfected into HEK293T cells in company with packaging and envelope plasmids (psPAX2, pMD2G) using PEI (Polysciences, Warrington, PA, United States). Virus were collected at 48 h post-transfection. After filtered, MEFs were infected with viruses plus 8 μg/mL polybrene (Sigma, St. Louis, MO, United States) 24 h for overexpression or 48 h for gene knockdown before analysis.

### RNA Sequencing and Analysis

Two groups of MEFs (PRE and SEN, 2 × 10^6^ cells per group) were used to generate the RNA sequencing data. Total RNA and purified polysomal RNA from each group (Total PRE, Total SEN, Polysomal PRE, Polysomal SEN) were checked for a RIN number to inspect RNA integrity by an Agilent Bioanalyzer 2100 (Agilent, Santa Clara, CA, United States). Qualified RNA with a RIN number between seven and eight was further purified by RNeasy micro kit and RNase-Free DNase Set (QIAGEN, Hilden, Germany). Double-stranded cDNA was synthesized from input of 1 μg RNA of each sample by SuperScript II Reverse Transcriptase (Invitrogen, Waltham, MA, United States) and isolated by Ampure XP beads (Beckman, Atlanta, GA, United States). A tailing mix was added to each sample and followed with adapter ligated and PCR amplification following the TruSeq Stranded mRNA LT Sample Preparation Kit Guide (Illumina 15032612, San Diego, CA, United States). Each sample without biological replication was made into one library, which were further quantified by Qubit 2.0 Fluorometer (Invitrogen, Waltham, MA, United States) and verified by Agilent 2100 bioanalyzer. Four cDNA libraries were sequenced using HiSeq 2500 system (Illumina, San Diego, CA, United States) in a mode of 150-bp paired-end read from Shanghai Biotechnology Corporation, generating raw data from 6.69 to 10.44 Gb for each sample.

To perform differential analysis, clean reads of each sample that mapped to mouse reference genome (GRCm38/mm10) with clean ratios above 94.96% were obtained using HISAT2 (v2.0.4) (Kim et al., 2015). Sequencing read counts were calculated using StringTie (v1.3.0) (Pertea et al., 2015). The expression levels of different samples were normalized by the Trimmed Mean of *M* values (TMM) method (Robinson and Oshlack, 2010) and converted into FPKM. The edgeR package of R was used to analyze the difference gene expression between samples (Robinson et al., 2010). The *p*-value was calculated and the multiple hypothesis test was performed. Significantly different genes were defined as the ratio of FPKM is greater than 1.5 and *p*-value less than 0.05.

Volcano plot was generated by Perseus. The raw RNA-seq data have been submitted to SRA database with BioProject accession number of PRJNA573538.

### Statistical Analyses

Data were analyzed using two-tailed Student’s *t* test and expressed as mean ± SEM. Statistical significance was set at *p* < 0.05.

## Data Availability Statement

The datasets generated for this study can be found in the SRA database with BioProject accession number of PRJNA573538.

## Author Contributions

SX wrote the first draft of the manuscript. SW, SX, and RL conducted the experiments. KL, XZ, YYL, ZZ, YL, RF, and JZ discussed the manuscript and performed the parts of experiments. SW, ZS, and FL wrote the final version of the manuscript. All authors approved the final manuscript.

## Conflict of Interest

The authors declare that the research was conducted in the absence of any commercial or financial relationships that could be construed as a potential conflict of interest.

## Footnotes

1 https://www.ncbi.nlm.nih.gov/refseq/

## Abbreviations

4E-BP1: eukaryotic translation initiation factor 4E binding protein 1
Cacul1, CDK2 associated: cullin domain 1
eIF4B: eukaryotic translation initiation factor 4B
FPKM: fragments per kilobase of transcript per million mapped fragments
GO: gene ontology
Haus1, HAUS augmin-like complex: subunit 1
Log: logarithm
MEF: mouse embryonic fibroblast
mTORC1: Mammalian/mechanistic target of rapamycin complex 1
PD: population doubling
PRE: presenescent
ROS: reactive oxygen species
RT-qPCR: reverse transcription-quantitative polymerase chain reaction
S6K: p70 S6 kinase 1
S6K1: ribosomal protein S6 kinase 1
SEN: senescent
SILAC: stable isotope-labelling with amino acids in cell culture
SUnSET: surface sensing of translation
TOP: 5 ′ terminal oligopyrimidine structure
Trdmt1: tRNA aspartic acid methyltransferase 1
TSS: transcription start site
Zfp280d: Zinc finger protein 280D.
