# Supplementary material for: mTORC1-Rps15 Axis Contributes to the Mechanisms Underlying Global Translation Reduction During Senescence of Mouse Embryonic Fibroblasts
Source: Front Cell Dev Biol. 2019 Dec 11;7:337. doi: 10.3389/fcell.2019.00337 (PMC6917584; doi:10.3389/fcell.2019.00337)
Supplement: Supplementary file 1 [file Data_Sheet_1.PDF]

frontiers

Supplementary Material

1 Supplementary Data

Supplementary Material should be uploaded separately on submission. Please include any supplementary data, figures and/or tables. All supplementary files are deposited to FigShare for permanent storage and receive a DOI.

2 Supplementary Figures and Tables

2.1 Supplementary Figures

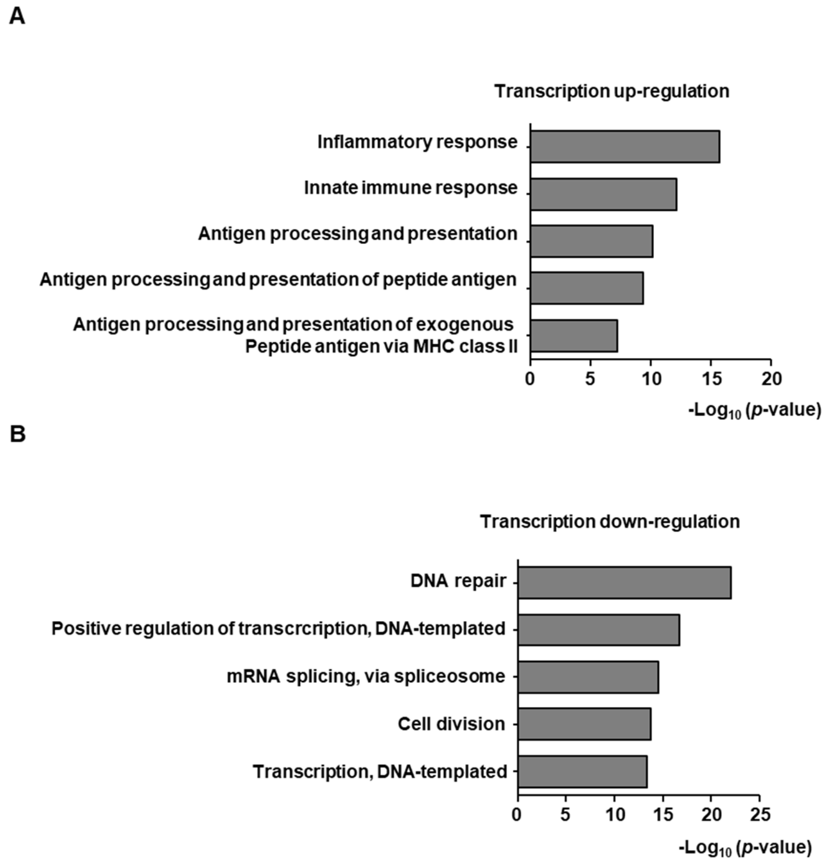

Supplementary Figure S1. GO biological process analysis of RNA-seq data of total mRNA fractions. **(A)** Analysis of transcriptional up-regulated genes. **(B)** Analysis of transcriptional down-regulated genes.

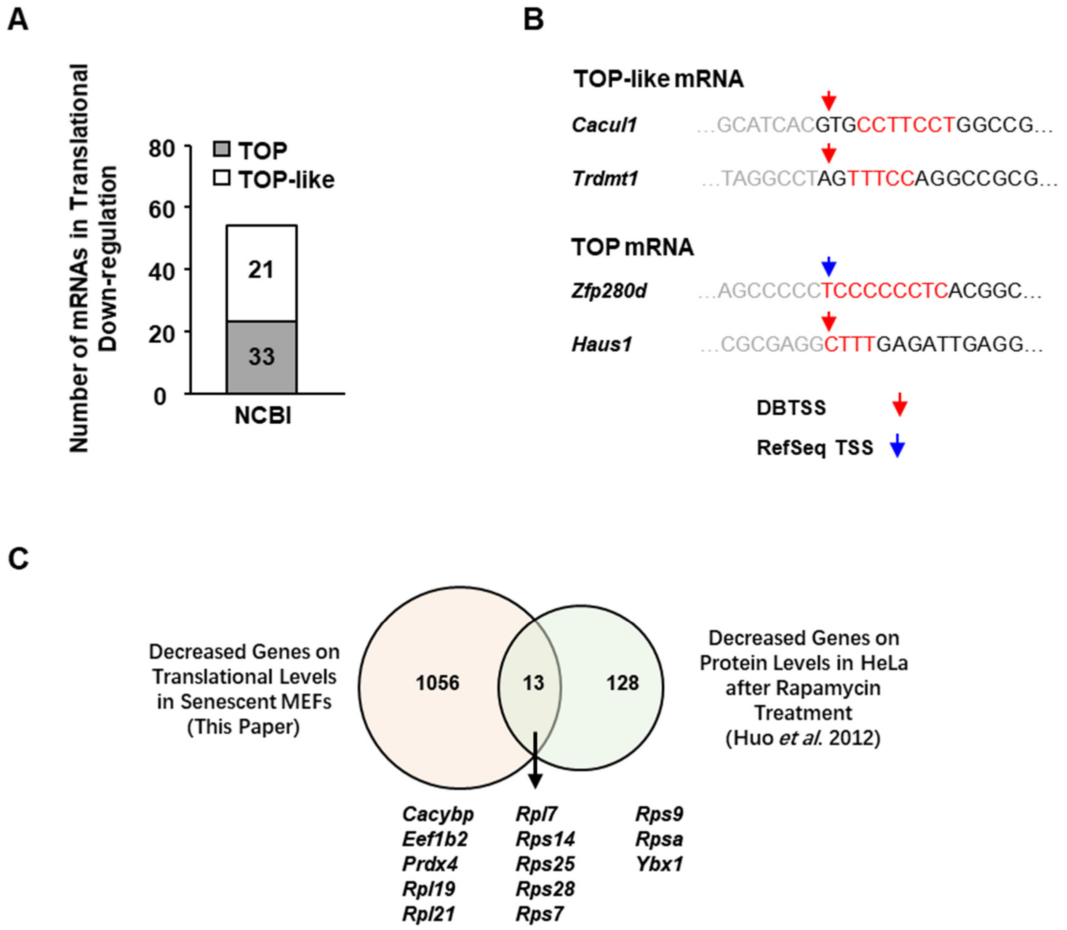

Supplementary Figure S2. Translationally downregulated candidate genes with TOP or TOP-like structures in RNA-seq results. (A) The number of translationally down-regulated mRNAs which contain TOP or TOP-like structures. **(B)** Transcription start site annotations for candidate mRNAs from DBTSS (https://dbtss.hgc.jp/) or Refseq database. Arrow head indicates transcription start site. Red

letters refer to TOP or TOP-like structures. **(C)** Venn diagram shows the comparison of translationally decreased genes between this study and Huo *et al.* 2012.

A

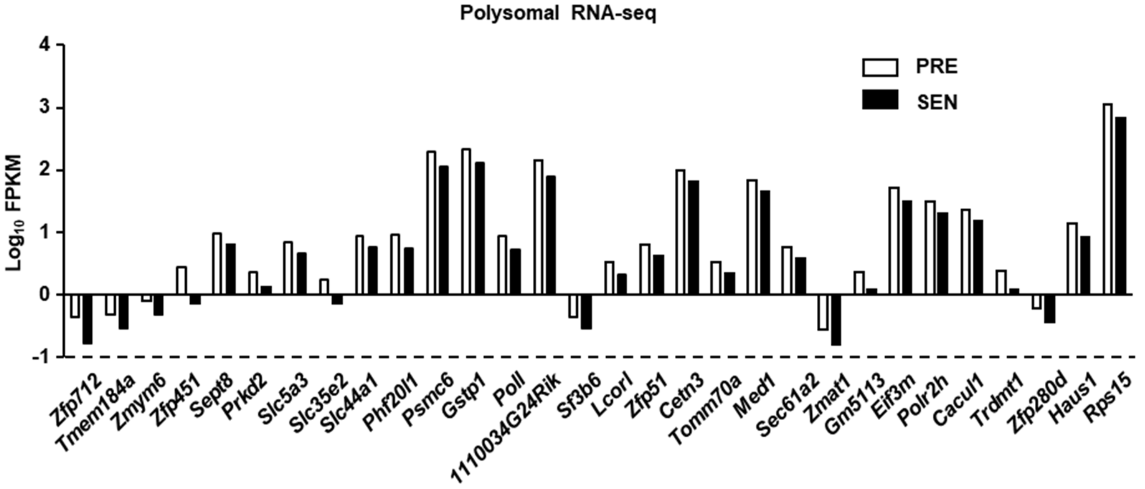

B

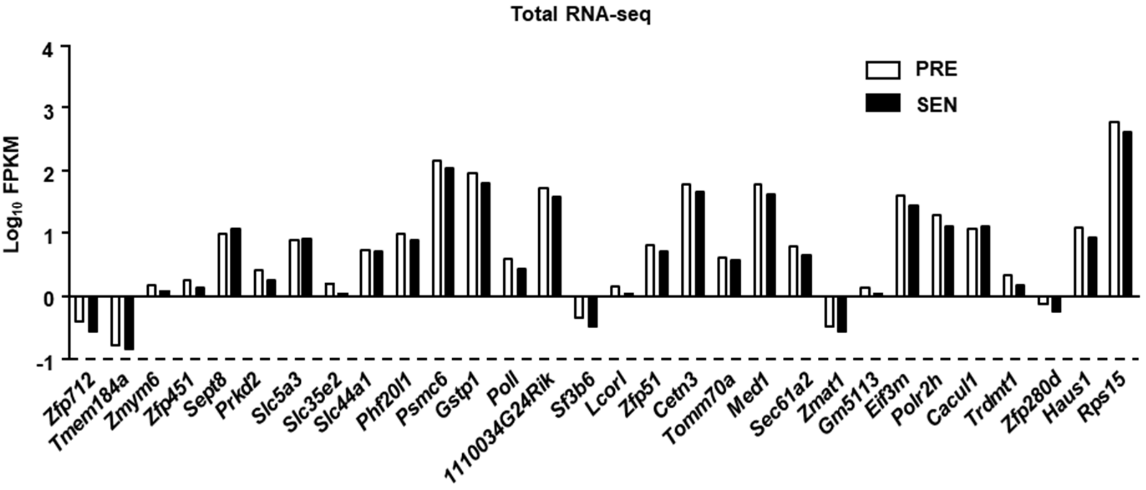

Supplementary Figure S3. RNA-seq results of TOP or TOP-like candidate genes for further PCR confirmation. (A) FPKM value of 30 candidate genes for further PCR confirmation in the

polysomal fractions of PRE and SEN group. **(B)** FPKM value of 30 candidate genes for further PCR confirmation in the total fractions of PRE and SEN group.

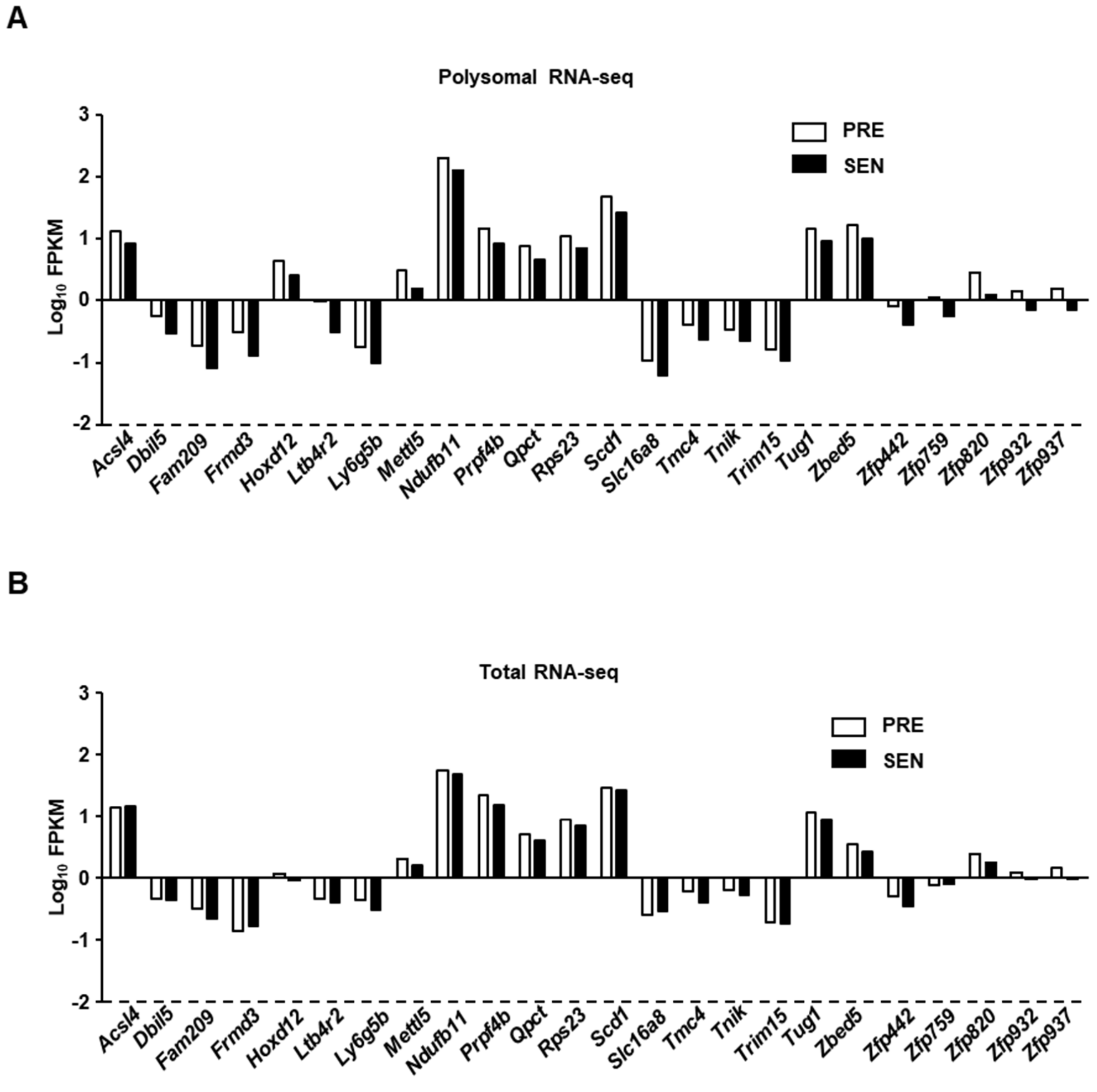

Supplementary Figure S4. RNA-seq results of TOP or TOP-like candidate genes without further PCR confirmation. (A) FPKM value of 24 candidate genes without further PCR

confirmation in the polysomal fractions of PRE and SEN group. **(B)** FPKM value of 24 candidate genes without further PCR confirmation in the total fractions of PRE and SEN group.

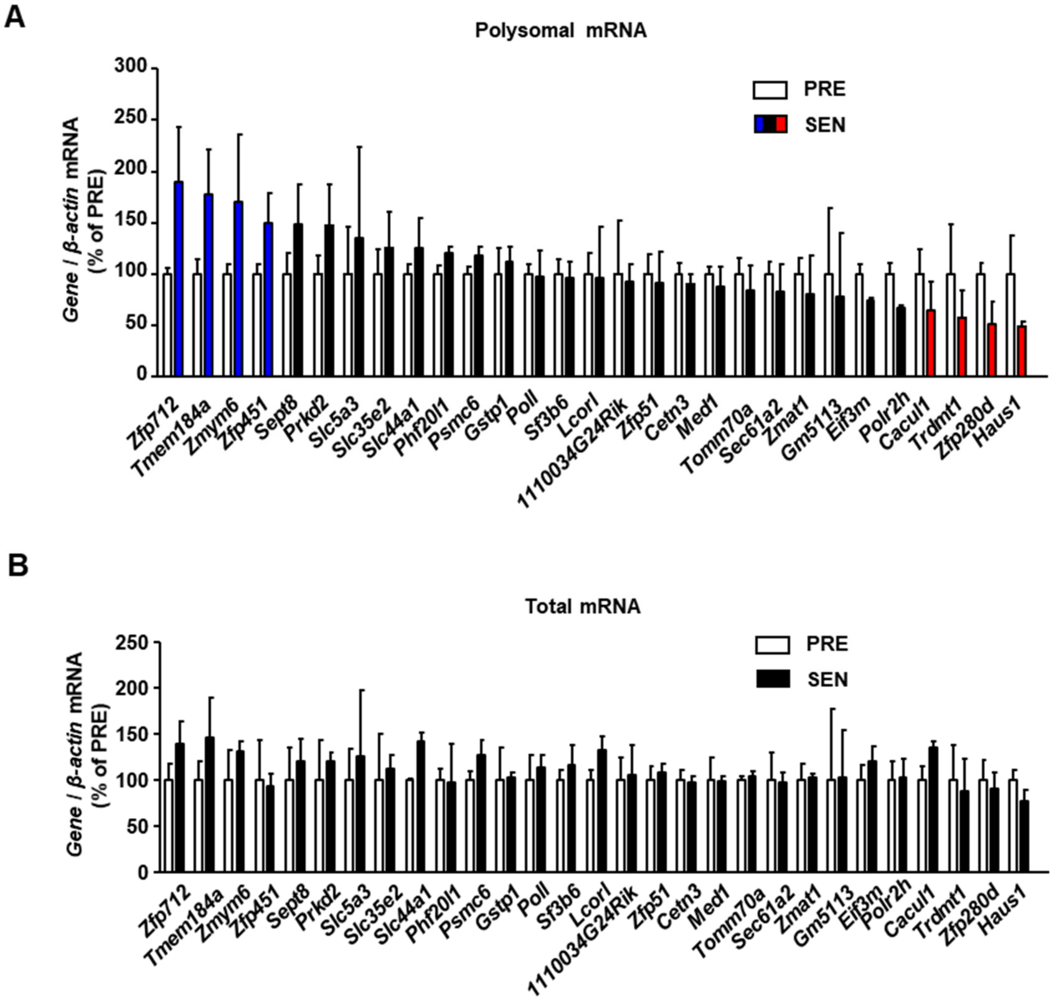

Supplementary Figure S5. RT-qPCR screen of candidate genes of which translation is down-regulated during MEF senescence. (A-B) Relative quantification of candidate mRNAs using

polysomal mRNAs **(A)** and total mRNAs **(B)** extracted from young and senescent MEFs. β-actin was used as internal control. (Mean±SEM, n=3).

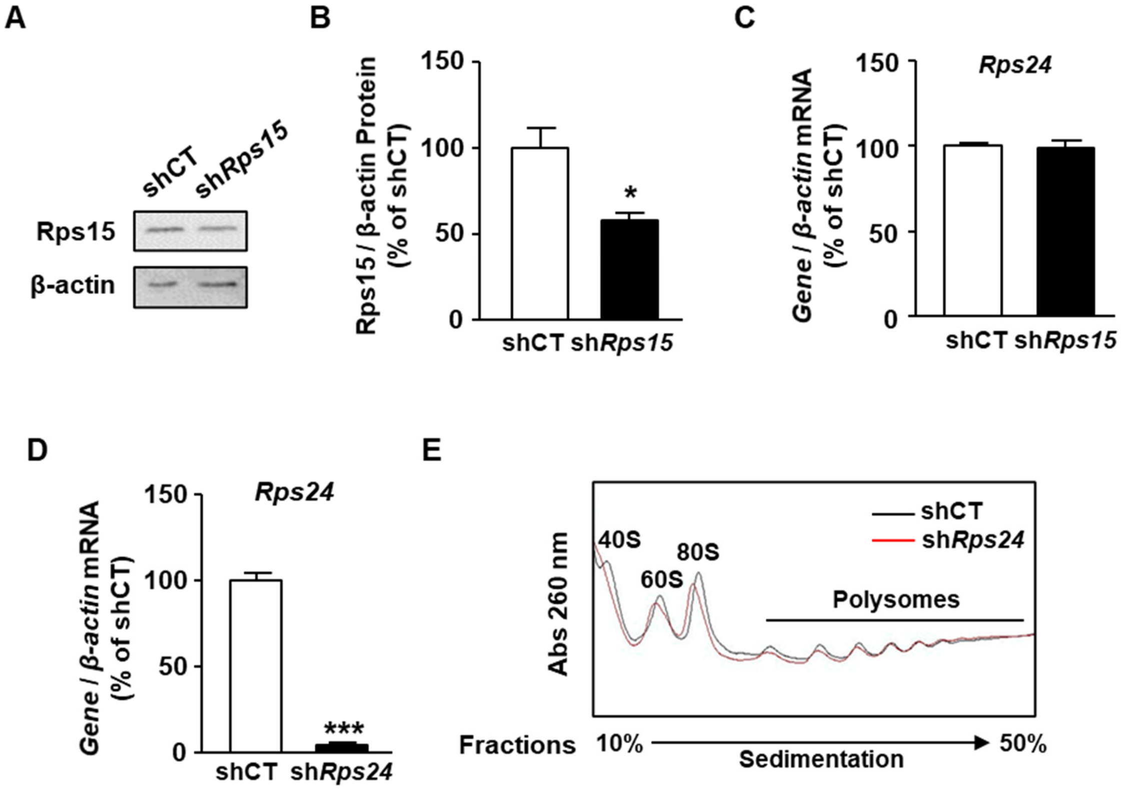

Supplementary Figure S6. ***Rps24*** **might not be a potential target as** ***Rps15*** **in regulating senescence of MEFs. (A-B)** Representative western blot and quantification of Rps15 protein level in cell extracts from control and Rps15-knockdown MEFs. β-actin were used as internal loading control. (Mean±SEM, n=3, *p<0.05). **(C-D)** Relative RT-qPCR quantification of *Rps24* mRNA extracted from control and *Rps15*-knockdown MEFs **(C)** or *Rps24*-knockdown MEFs **(D)**. *β-actin* was used as internal control. (Mean±SEM, n=3, ***p<0.001). **(E)** Polysomal profiles of sh*Rps24* MEFs and negative control with continuous sucrose gradient of 10%-50% were fractioned and

measured with absorbance of light at 260 nm. Peaks belonged to large subunit of 60S, intact ribosome of 80S and polysomes were labeled.

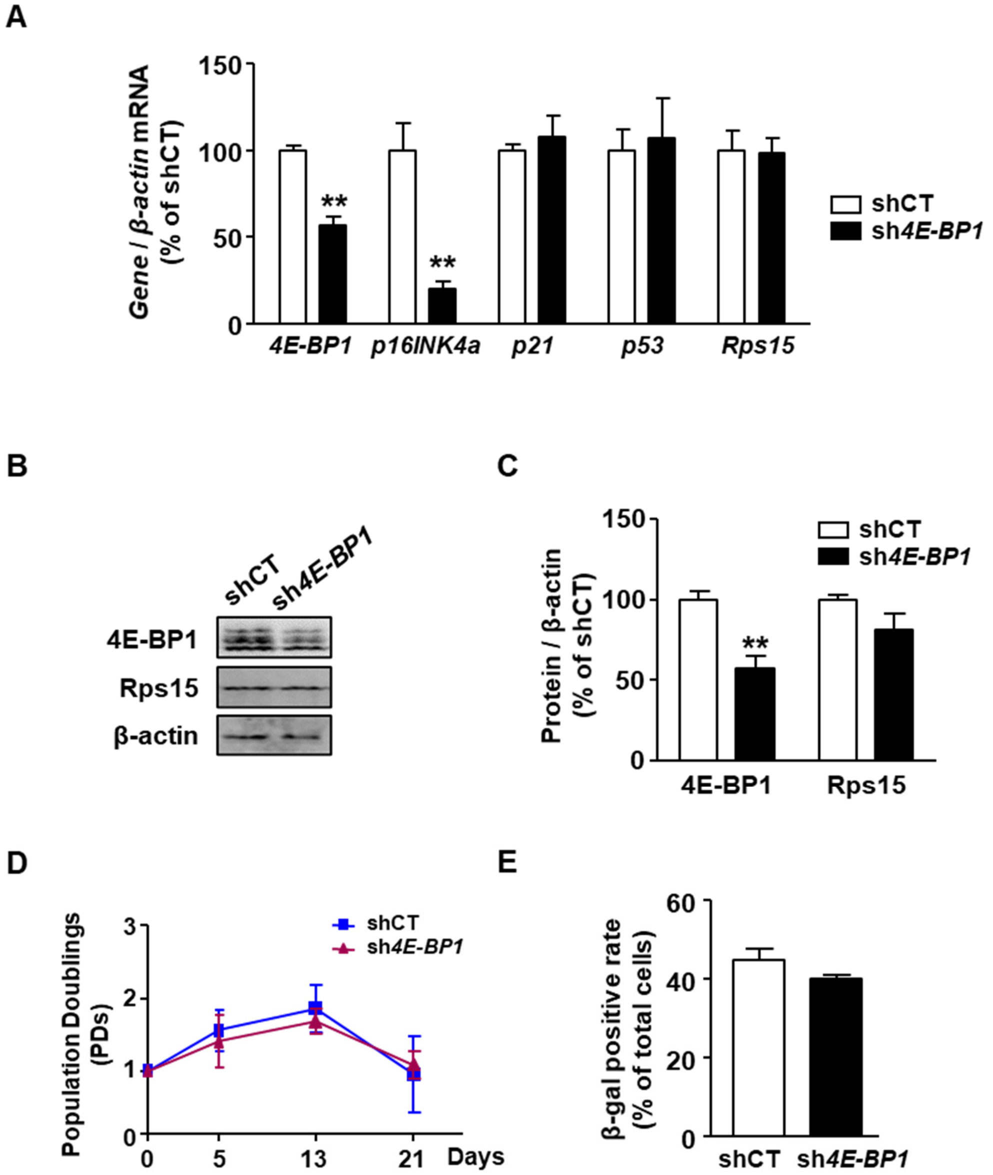

Supplementary Figure S7. Knockdown of *4E-BP1* cannot rescue MEF senescent phenotypes. **(A)** Relative RT-qPCR quantification of indicated mRNAs extracted from control and *4E-BP1*-knockdown MEFs. *β-actin* was used as internal control. (Mean±SEM, n=3, ***p*<0.01). **(B-C)** Representative western blot and quantification of Rps15 and 4E-BP1 protein level in cell extracts from control and *4E-BP1*-knockdown MEFs. β-actin were used as internal loading control. (Mean±SEM, n=3, **p*<0.05). **(D)** Growth curves of control and sh*4E-BP1* stable knockdown MEFs

starting from passage 1. (Mean±SEM, n=3). **(E)** SA-β-gal positive rate of control and sh*4E-BP1* stable knockdown MEFs. (Mean±SEM, n=3).

2.2 Supplementary Tables

Supplementary Table S1. Transcriptional Regulation.

Supplementary Table S2. Translational Regulation.

Supplementary Table S3. TOP or TOP-like mRNA in RNA-seq.

Supplementary Table S4. Primers for Q-PCR analysis.
