# Supplementary material for: mTORC1-Rps15 Axis Contributes to the Mechanisms Underlying Global Translation Reduction During Senescence of Mouse Embryonic Fibroblasts
Source: Front Cell Dev Biol. 2019 Dec 11;7:337. doi: 10.3389/fcell.2019.00337 (PMC6917584; doi:10.3389/fcell.2019.00337)
Supplement: Supplementary file 5 [file Table_4.docx]

**Supplementary Table 4: Primers for Q-PCR analysis.**

| **Gene Name** | **Forward Primer** | **Reverse Primer** |
| --- | --- | --- |
| *mZfp712* | 5'-TCTCTAAGCCGTACTTGGTCA-3' | 5'-TTGCTCCAGGACGAATTTTATGT-3' |
| *mTmem184a* | 5'-CCATCTACGCCTTCGACTCC-3' | 5'-TTCGTAGCAGTCTCGCACAG-3' |
| *mZmym6* | 5'-ACAAAGCAATGGCACAACAA-3' | 5'-GACGGTAGACCAACCTGAAA-3' |
| *mZfp451* | 5'-GCACGCTCATGGTTTACAAGA-3' | 5'-GCACGCTCATGGTTTACAAGA-3' |
| *mSept8* | 5'-AGTTTCCCACCGATGACGAG-3' | 5'-CCCAAGGGTACTGTCGTGC-3' |
| *mPrkd2* | 5'-GGGGTCTCCTTCCATATCCAG-3' | 5'-CACGATAGAACAGGCTAGTTGC-3' |
| *mSlc5a3* | 5'-TTGCCGTAGTGGCCCTGTA-3' | 5'-GCCAGCCCAATGAAGTGTTC-3' |
| *mSlc35e2* | 5'-CAGCAGCGAAATCCCAAGTG-3' | 5'-CTTCGGTGACCAAATAGAGACC-3' |
| *mSlc44a1* | 5'-AAATCCTGCATTTGCTGCCTT-3' | 5'-GCGTTCTCCACTAGAATGACGAA-3' |
| *mPhf20l1* | 5'-TGAAGCTGTCATTAGCAGTTCTG-3' | 5'-CAAGTGGTGACTTAGGGGTTTT-3' |
| *mPsmc6* | 5'-CCTGTCAGAACAGATTCGGGA-3' | 5'-GCAAACAGCCTTTTGGAGGTATT-3' |
| *mGstp1* | 5'-ATGCCACCATACACCATTGTC-3' | 5'-GGGAGCTGCCCATACAGAC-3' |
| *mPoll* | 5'-GACAGACACGGAAGGATTCAG-3' | 5'-GAAAGGGTGGTCCTGGGTAAA-3' |
| *mSf3b6* | 5'-ATGGACCTATTCGTCAAATCAGAGT-3' | 5'-CGCAGGCATTCTTAGCATCAA-3' |
| *mLcorl* | 5'-ATGCCATAAAGTATGCCTGCTT-3' | 5'-ATGCCATAAAGTATGCCTGCTT-3' |
| *m1110034G24Rik* | 5'-GGCCAGTTGGAGACCAATGA-3' | 5'-CACAGATGCAGAGAGCGGAT-3' |
| *mZfp51* | 5'-AGCATCACAAGCTGGAGAGTC-3' | 5'-CCTGGGGCATGTTTACTGGT-3' |
| *mCetn3* | 5'-CTGAGAGGTGAGCTTGTAGTAGA-3' | 5'-TTGGTCTTTGTCGGTATCAAACA-3' |
| *mMed1* | 5'-GAGTGATGACTGCCCACCAA-3' | 5'-CACTATTGGGGCTTCCAGCA-3' |
| *mTomm70a* | 5'-AGCAAGCTATTCAGTGCTACAC-3' | 5'-AGGGCTTTCACATATTTGGGATT-3' |
| *mSec61a2* | 5'-ATTTTCAGGGGTTTCGTGTTGA-3' | 5'-GCCACTAAATCGAACAGACAGC-3' |
| *mZmat1* | 5'-AAGCCCAAGCCCATGAATACA-3' | 5'-ACTCTGCTGGTAAATTGTGAGG-3' |
| *mGm5113* | 5'-ACATCAGCCATCAGAGCGTC-3' | 5'-ACGAACTACGACTTTCCACAG-3' |
| *mEif3m* | 5'-TGGGATGGACAAGAATACTCCT-3' | 5'-GATCCAGCTCCGTTGGGATG-3' |
| *mPolr2h* | 5'-TGCACTGTGAGAGTGAATCTTTC-3' | 5'-CGGAACTTGTCACCTAAGTCC-3' |
| *mCacul1* | 5'-ACTTTCTGAGTACGCTGCCC-3' | 5'-TTCCGGGACTGATCACCTCT-3' |
| *mRps15* | 5'-ACCTACCGTGGCGTAGACC-3' | 5'-TGTCCCTCAGGTGCGTCTT-3' |
| *mTrdmt1* | 5'-TGGACCACGTATCTGTGCTG-3' | 5'-CGCAGCAATAACTTGGGTGG-3' |
| *mZfp280d* | 5'-CGTTGCACCAAATGCAGACT-3' | 5'-GGTAGAAGTGCTTGGGCTGA-3' |
| *mHaus1* | 5'-GGACCCTGTTGCGTTCTGAA-3' | 5'-GGACCCTGTTGCGTTCTGAA-3' |
| *mActin* | 5'-CACTGTGCCCATCTACGA-3' | 5'-CAGGATTCCATACCCAAG-3' |
| *mp16INK4a* | 5'-CCCAACGCCCCGAACT-3' | 5'-GCAGAAGAGCTGCTACGTGAA-3' |
| *mp21* | 5’-CCTGGTGATGTCCGACCTG-3’ | 5’-CCATGAGCGCATCGCAATC-3’ |
| *mp53* | 5’-CTCTCCCCCGCAAAAGAAAAA | 5’-CGGAACATCTCGAAGCGTTTA-3’ |
| *m4E-BP1* | 5'-CCGGGAGGAACCAGGATTAT-3' | 5'-TAGTGACCCCAGGAATGGCT-3' |
| *mRps24* | 5'-AGTGAGCGGTCCTCTTTTCC-3' | 5'-GAACTTCCTGGTCCGGATGG-3' |
| *Spike mScarlet* | 5'-CGTGCTGAAGGGCGACATTA-3' | 5'-CGGTGTAGTCCTCGTTGTGG-3' |
